# iNKT Cells Control Mouse Spontaneous Carcinoma Independently of Tumor-Specific Cytotoxic T Cells

**DOI:** 10.1371/journal.pone.0008646

**Published:** 2010-01-13

**Authors:** Matteo Bellone, Monica Ceccon, Matteo Grioni, Elena Jachetti, Arianna Calcinotto, Anna Napolitano, Massimo Freschi, Giulia Casorati, Paolo Dellabona

**Affiliations:** 1 Cellular Immunology Unit, Division of Immunology, Transplantation and Infectious Diseases, Istituto Scientifico San Raffaele, Milan, Italy; 2 Experimental Immunology Unit, Division of Immunology, Transplantation and Infectious Diseases, Istituto Scientifico San Raffaele, Milan, Italy; 3 Unità Operativa Anatomia Patologica, Istituto Scientifico San Raffaele, Milan, Italy; 4 Università Vita Salute San Raffaele, Milan, Italy; New York University, United States of America

## Abstract

**Background:**

CD1d-restricted invariant NKT (iNKT) cells are a subset of T lymphocytes endowed with innate effector functions that aid in the establishment of adaptive T and B cell immune responses. iNKT cells have been shown to play a spontaneous protective role against experimental tumors. Yet, the interplay between iNKT and tumor-specific T cells in cancer immune surveillance/editing has never been addressed. The transgenic adenocarcinoma of the mouse prostate (TRAMP) is a realistic model of spontaneous oncogenesis, in which the tumor-specific cytotoxic T cell (CTL) response undergoes full tolerance upon disease progression.

**Principal Findings:**

We report here that lack of iNKT cells in TRAMP mice resulted in the appearance of more precocious and aggressive tumors that significantly reduced animal survival. TRAMP mice bearing or lacking iNKT cells responded similarly to a tumor-specific vaccination and developed tolerance to a tumor-associated antigen at comparable rate.

**Conclusions:**

Hence, our data argue for a critical role of iNKT cells in the immune surveillance of carcinoma that is independent of tumor-specific CTL.

## Introduction

Several lines of evidence in pre-clinical and clinical studies support the notion that tumor cell growth is under the active control of the immune system [1]. Cancer immune surveillance is the result of concerted actions between innate and adaptive immune responses [2]. T cell responses specific for tumor-associated antigens (TAA) presented by MHC molecules on cancer cells play a central role in cancer immune surveillance, and can be increased by different immunotherapy strategies in attempts to cure cancer [3].

iNKT cells are a subset of conserved T lymphocytes that bridge innate and adaptive immunity [4]. They are characterized by the expression of the homologous invariant (i)Vα14-Jα18 and Vα24-Jα18 TCR chains in mice and humans, respectively [5], [6]. The iVα chains pair with a set of variable TCRβ chains exhibiting a very restricted Vβ gene usage: Vβ8.2, 7 and 2 in mice and Vβ11 in humans [4]. This semi-invariant TCR recognizes endogenous or exogenous lipid antigens (Ag) presented by the MHC-class I like molecule CD1d [7].

Unlike MHC-restricted T cells, iNKT cells exhibit an effector-memory phenotype independently of foreign Ag encounter, acquired in the thymus following a distinct developmental pathway [8]. Injection into mice of the CD1d-restricted glycosphingolipid Ag αGalactosylCeramide (αGalCer) potently activates iNKT cells, triggering within hours copious production of a wide range of Th1 and Th2 cytokines [9]. These cytokines, in turn, activate effector cells of both innate and adaptive immune responses [9]. iNKT cell interaction with CD1d-expressing immature dendritic cells (DC) results in the licensing of antigen presenting functions, which facilitates priming of CD4^+^ and CD8^+^ T cell and B cell responses specific for concomitant protein Ag [10], [11]. Furthermore, cytokine production by iNKT cells can be triggered independently of TCR engagement, for instance by the action of inflammatory cytokines such as IL-12 and IL-18 [12].

Because of these peculiar innate-like effector characteristics, iNKT cells are regarded as potent adjuvant involved in the early activation of the immune response and in cancer immune surveillance [13], [14]. Consistent with this function, circulating iNKT cell number have been found reduced in human solid tumors, while IFN-γ production and number of iNKT cells appear to correlate with a more favorable prognosis in multiple myeloma, colorectal cancer, head and neck cancer and prostate cancer (PC) [15], [16], [17], [18].

iNKT cell activation by αGalCer *in vivo* results in the subsequent activation of NK and T cells, leading to the growth control of both transplantable tumors and spontaneous mammary carcinoma [19], [20], [21].

iNKT cells display also a spontaneous anti-tumor function, which occurs independently of αGalCer administration, possibly induced by the recognition of endogenous lipid antigens. Mice selectively deficient in iNKT cells (Jα18^−/−^ mice) are in fact significantly more susceptible to chemically induced carcinogenesis [22]. In this model, adoptive cell transfer experiments established that the protection from methylcholanthrene (MCA)-induced fibrosarcoma is mediated by IFN-γ-producing hepatic CD4^−^ iNKT cells [23]. Furthermore, iNKT cells spontaneously suppress the growth of osteosarcoma and hematopoietic tumors caused in mice by the loss of the tumor suppressor p53 [24]. Because of the lack of a traceable tumor Ag-specific T cell response in both MCA-induced tumor models and in p53^+/−^ mice, and development of different and unpredictable tumor types in the latter, it is difficult to correlate the presence or lack of iNKT with tumor-specific a CTL response and its involvement in cancer immune surveillance.

Transgenic adenocarcinoma of the mouse prostate (TRAMP) mice express the SV40 large T antigen (Tag) in the prostate epithelium under the control of the rat probasin regulatory element. Sexual hormones influence the expression of the transgene [25]; hence, male mice remain healthy until puberty. In the following weeks, TRAMP mice over-express Tag and invariably develop spontaneous mouse prostate intraepithelial neoplasia (mPIN), which progress to adenocarcinoma and seminal vesicles, lymph node (LN) and visceral metastases, resembling human PC [26]. In TRAMP mice, the immune response against the surrogate tissue-specific TAA Tag is characterized by thymic deletion of high avidity CTL [27]. As a consequence, vaccination with Tag-pulsed DC in young healthy TRAMP mice elicits low avidity CTL specific for the immunodominant sequence Tag_404–411_ (Tag-IV) [28]. In parallel with PC development and progression, these CTL undergo a profound state of peripheral tolerance [29] that cannot be rescued by DC vaccination [29], [30]. A similar phenomenon has been reported in TRAMP mice for other surrogate [31] and natural tumor associated antigens [32], and recapitulates the tolerant status found in patients with advanced PC disease [33].

Given the data on anti-tumor roles of NKT cells in transplantable and carcinogen tumor models together with the clinical relevance of the TRAMP model, we investigated whether iNKT cells control the growth of spontaneous PC, and whether this phenomenon depends on the interplay between iNKT and tumor-specific T cells.

## Materials and Methods

### Mice, Tumor Cell Lines and Reagents

Heterozygous TRAMP mice on a C57BL/6 background were obtained from Dr. A Vitiello (The R.W. Johnson Pharmaceutical Research Institute, San Diego, CA), originated from the founder male 8247 [25], and were bred by crossing heterozygous TRAMP females with wild type (WT) C57BL/6 males. B6.129-Tcra-Jtm1tg mice Jα18-deficient (Jα18^−/−^) were obtained from Dr. M. Taniguchi (RIKEN, Yokohama, Japan) and backcrossed 12 times to C57BL/6. TRAMPJα18^−/−^ mice were generated at San Raffaele by crossing TRAMP and C57BL/6 Jα18^−/−^ mice. Animals were typed for Tag expression by PCR-based screening assay, as described in (www.jax.org). All mice were housed and bred in a specific pathogen free animal facility. All procedures involving animals were reviewed and approved by the Institutional Animal Care and Use Committee at San Raffaele Scientific Institute. Animals were monitored twice a week for weight and tumor growth, and euthanized when signs of bulky prostate tumor and/or distress were evident. RMA is a H-2^b^ Rauscher virus-induced thymoma [34]. B6/K-0 is a kidney cell line, expressing Tag [35]. TRAMP-C1 is a PC cell line originated from a TRAMP mouse [36]. Unless specified, all chemical reagents were from Sigma-Aldrich, and mAb were from BD PharMingen (San Diego, CA).

### DC Preparation and Immunization Protocols

DC were prepared and characterized as previously described [37]. DC were pulsed with 2 µM Tag-IV or 1 µM tyrosinase related protein 2 peptide_181–200_ (TRP-2; Research Genetics, Huntsville, AL) for 1 h at 37°C, washed, and suspended at 1×10^6^/ml in PBS. Five×10^5^ DC were injected i.d. into mice. When requested, αGalCer (100 ng/ml) was added to the DC cultures 3 h before cell recovery. Magnetic-bead (Miltenyi Biotec, Bologna, Italy) enriched CD8^+^ splenocytes [38] from mice euthanized one week after DC vaccination were stained with PE-labeled K^b^/Tag-IV or K^b^/OVA (SIINFEKL) pentamers (ProImmune, Oxford, UK), CD8, CD44, and dump (B220, CD11c, CD19, CD4) mAbs and analyzed by flow cytometry [29]. For functional analysis of iNKT cells, αGalCer (2 µg/mouse; Alexis, Lausen, Switzerland) was administered i.v. Blood samples were collected from the tail vein 2, 6 and 24 hours later, and the serum content of IL-4 and IFN-γ was determined by standard ELISA (BD Pharmingen, San Diego, CA). Animals were euthanized 72 hours after αGalCer treatment, and the number of iNKT cells in their spleen and liver was determined by staining with αGalCer-mCD1d tetramers (provided by the NIH Tetramer Facility) and anti-TCRβ mAbs and flow cytometry analysis [39].

### In Vitro Cytotoxicity Assay

Splenocytes were re-stimulated *in vitro* in the presence of 1 µM Tag IV or TRP-2 peptide. Day-5-blasts were tested for cytolytic activity in a standard 4 h ^51^Cr release assay [37]. ^51^Cr release of target cells alone was always <25% of maximal ^51^Cr release (target cells in 0.25 M SDS).

### Histology and Immunohistochemistry

The urogenital apparatus (UGA) was excised, weighted, fixed in 4% formalin for 6 h, then embedded and included in paraffin wax. H&E and Tag staining of 5-mm thick sections were performed as previously described [30]. Macroscopic and microscopic specimens were evaluated by a pathologist in a blind fashion. Histology sections were scored as previously described [30], [40] with partial modifications: 0, healthy tissue; <1, scattered (<20%) Tag^+^ cells with mild increase of nucleus to cytoplasm ratio; 1, ≥20% Tag^+^ cells with increasing nucleus to cytoplasm ratio, nuclear hyperchromasia, stratification and micro-papillary projections; 2, presence of all the features described in 1 and cribriform structures with mild enlargement of acini; 3, presence of all the features described in 2 and either proliferation of the epithelial and stromal cells of the seminal vesicles or mild proliferation of smooth muscle stromal cells of prostatic acini with acinar enlargement; 4, presence of all the features described in 3 and marked proliferation of smooth muscle stromal cells with penetration of malignant Tag^+^ cells through the basement membrane of mPIN-involved glands into the surrounding stroma; 5, well differentiated adenocarcinoma; and 6, presence of metastasis and/or neuroendocrine tumors.

### Statistical Analysis

Statistical analyses were performed using the Student *t*-test, the Log-Rank and the Mann-Whitney tests. Comparison of survival curves was considered statistically significant for p<0.05.

## Results

### Survival of TRAMP Mice Lacking iNKT Cells Is Reduced

**Figure 1:**
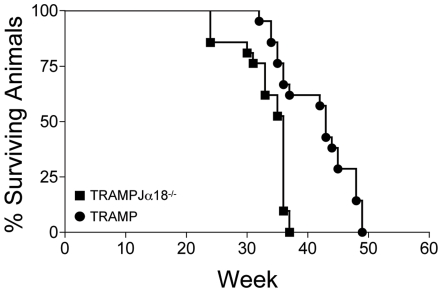
Survival of TRAMP mice lacking iNKT cells is reduced. Kaplan-Maier plot reporting the survival curves of groups of male TRAMP (black squares; n = 21) and TRAMPJα18^−/−^ mice (black circles; n = 21). Animals were examined twice a week and euthanized when signs of bulky prostate tumor and/or distress were manifest. At necropsy, the anatomy and histology of the UGA was analyzed as indicated in the Material and Methods section. Animals were attributed a disease score≥4. Statistical comparison (Log-Rank test) between the survival curves: p<0.0001.

To investigate the role of iNKT cells in immune surveillance against a spontaneous solid cancer, we generated male TRAMPJα18^−/−^ mice, and compared their survival with a cohort of TRAMP mice. Animals were euthanized when signs of bulky prostate tumor and/or distress were manifest. Anatomy and histology of the UGA confirmed the presence of aggressive prostate tumors in all animals (i.e., disease score ≥4; data not shown). As reported in Fig. 1, TRAMPJα18^−/−^ mice exhibited a reduced disease-free survival, and all animals in this cohort were euthanized before week 38. At this time, approximately 60% of the TRAMP animals were still alive and apparently tumor free. Comparison of survival curves of the two cohorts of mice revealed a highly significant prolonged survival for TRAMP mice (p<0.0001). Hence, the lack of iNKT cells appears to greatly affect tumorigenesis in this model.

### Tumor Onset Is Accelerated in TRAMP Mice Lacking iNKT Cells

**Figure 2:**
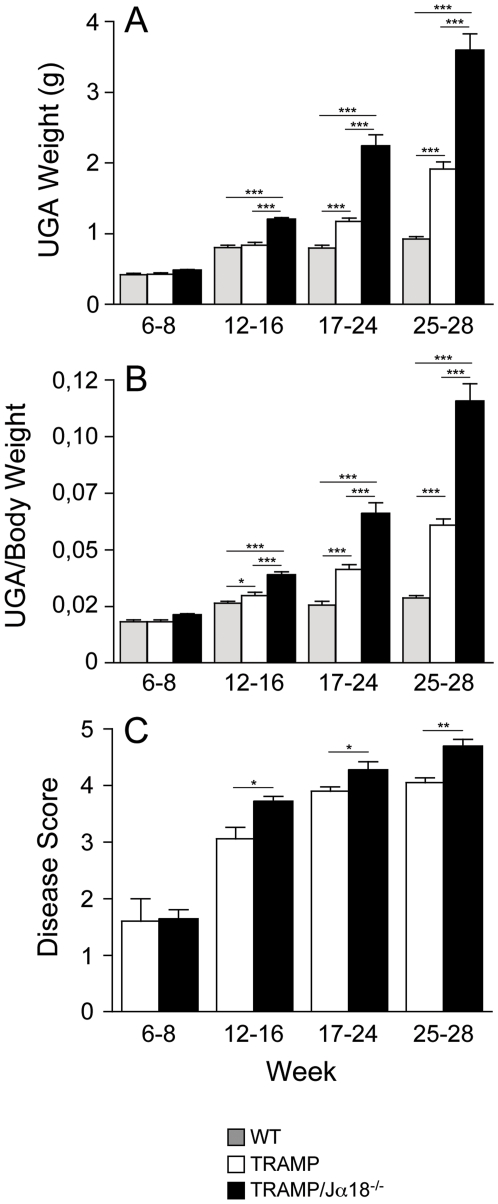
Tumor onset is accelerated in TRAMP mice lacking iNKT cells. Age- and sex-matched male TRAMP (white bars; n = 97), TRAMPJα18^−/−^ (black bars; n = 51) and WT mice (gray bars; n = 97) were killed at the indicated week after birth and the anatomy and histology of the UGA was analyzed as indicated in the Material and Methods section. Animals affected by neuroendocrine tumors were excluded. A, UGA weight (g), expressed as average±SEM, of TRAMP, TRAMPJα18^−/−^ and WT mice killed at weeks 6–8 (n = 5, 8 and 19, respectively), 12–16 (n = 16, 17 and 26), 17–24 (n = 30, 15 and 26) and 25–28 (n = 46, 11 and 26), respectively. B, ratio of the weight of UGA and body weight subtracted of the UGA in the groups of animals reported above. C, Disease score, expressed as average±SEM, of the groups of animals reported above. Statistical analysis of collected data was performed using the Student's *t*-test (A and B) and the Mann-Whitney test (C); ***p<0.001, **0.001<p<0.05, *0.01<p<0.05.

To better define the time of appearance and aggressiveness of prostate tumors in TRAMPJα18^−/−^ mice, cohorts of TRAMP and TRAMPJα18^−/−^ mice were sacrificed at different weeks after birth and full autopsy was performed. Animals with bulky and round-shaped prostate masses, which were histologically confirmed to be neuroendocrine prostate tumors, were excluded from the study because appearance of these lesions is stochastic in the TRAMP model [41]. Macroscopically, the UGA of TRAMPJα18^−/−^ mice appeared larger than that of age-matched WT and even TRAMP males, with seminal vesicles particularly engorged already at 17–18 weeks of age (data not shown). Indeed, the UGA weight, an accepted parameter to measure disease progression in this model, of TRAMP and TRAMPJα18^−/−^ males was higher than that of age-matched WT mice at weeks 17–24, and the difference further increased during the following weeks (Fig. 2A; p<0.0001). Furthermore, a highly statistically significant difference in UGA weight between TRAMPJα18^−/−^ and WT or TRAMP mice was present already at 12–16 weeks (p<0.0001), time at which there was no difference in UGA weight between TRAMP and WT males (Fig. 2A).

Difference in UGA weight was likely due to more advanced/aggressive tumors and not to a generalized increase in body weight of TRAMPJα18^−/−^ mice. Indeed, when the ratio of the weight of UGA and body weight subtracted of the UGA was calculated for all groups of animals reported above (Fig. 2A), differences were all confirmed (Fig. 2B).

Prostate tissues were also investigated microscopically. No difference in disease score was present in TRAMP and TRAMPJα18^−/−^ mice at 6–8 weeks of age, and most of the prostates at H&E staining showed scattered foci of cells with nuclear elongation, altered nucleus-to-cytoplasm ratio and micro-papillary projections, sometimes accompanied by cribriform structures and mild enlargement of acini (data not shown). Expression of Tag in 6–8 weeks old TRAMP mice and TRAMPJα18^−/−^ mice had a dim and patchy distribution overlapping pathologic foci (data not shown), with, as expected [30], a disease score<2 (Fig. 2C). In the following weeks, lesions in TRAMPJα18^−/−^ mice appeared more aggressive than that of age-matched TRAMP animals, and the disease score was significantly higher in TRAMPJα18^−/−^ than in TRAMP animals at all weeks tested (Fig. 2C).

Interestingly, invasive adenocarcinoma (disease score 5) was found in 4 out of 13 (31%) TRAMPJα18^−/−^ mice and in zero out of 8 18–19 week old TRAMP mice. In the TRAMP colony indeed, the earliest invasive adenocarcinoma was found at week 23. When cohorts of mice were sacrificed at week 25–28, adenocarcinoma was found in 7 out of 11 (64%) TRAMPJα18^−/−^ mice versus 7 out of 46 (15%) TRAMP mice.

Hence, spontaneous PC development and progression, as reported for tumors induced by MCA [22] or p53 loss [24], is more aggressive in TRAMP mice lacking iNKT cells.

### Similar Behavior of Tag-Specific CTL Responses in TRAMP and TRAMPJα18^−/−^ Mice

We next sought to investigate whether the iNKT cell-dependent anti-tumor functions could be related to the induction and maintenance of a spontaneous TAA-specific CTL response. The low frequency of tumor-specific CTL in TRAMP mice precluded the direct *ex vivo* comparison of frequency, phenotype and functions in TRAMP and TRAMPJα18^−/−^ mice [30]. However, the detection *ex vivo* of tumor-specific CTL responses can be achieved following *in vivo* expansion, obtained upon immunization of TRAMP mice with specific TAA. By immunizing TRAMP mice with DC pulsed with the TAA Tag, we were in fact able to detect a specific CTL response. Furthermore, we previously showed that PC growth is accompanied by the progressive induction of a Tag-specific CTL tolerance that reaches a full state at around 11 weeks of age [29], [30].

To determine whether the precocious and more aggressive PC found in TRAMPJα18^−/−^ was due to the establishment of Tag-specific CTL tolerance at an earlier stage than in TRAMP mice, TRAMP, TRAMPJα18^−/−^ and WT mice were immunized once i.d. with Tag-IV pulsed DC at week 6–8, when tumor-specific CTL are still functional, and sacrificed one week later. *Ex vivo* flow-cytometry analysis showed a comparable frequency of CD8^+^CD44^+^K^b^/TagIV^+^ cells in the spleen of vaccinated TRAMP and TRAMPJα18^−/−^ mice (0.9±0.14 and 1.31±0.5%, respectively), therefore indicating a similar *in vivo* expansion of Tag-specific T cells in TRAMP and TRAMPJα18^−/−^ mice.

**Figure 3:**
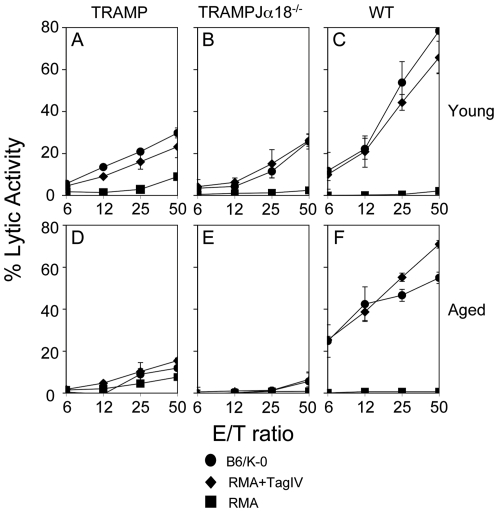
The immune response against Tag is comparable in TRAMP and TRAMPJα18^−/−^ mice. Tag-IV-pulsed DC were injected once i.d. into 6- (panels A–C) and 16-week (D–F) old male TRAMP (A and D), TRAMPJα18^−/−^ (B and E) and WT age- and sex-matched littermates (C and F). After 7 days, animals were killed and their splenocytes were stimulated *in vitro* with irradiated B6/K-0 cells, and tested 5 days later for cytotoxic activity (measured as ^51^Cr release); un-pulsed (black squares) or Tag-IV-pulsed (black diamonds) RMA and B6/K-0 (black circles) cells were used as targets. Data correspond to one out of at least three independent experiments, which gave similar results.

Upon in vitro Ag-specific restimulation, splenocytes from TRAMP and TRAMPJα18^−/−^ demonstrated a comparable, consistent and specific cytolytic activity against Tag-IV-pulsed RMA cells (Fig. 3A and B, respectively) that was, as expected [30], lower than the one found in WT vaccinated mice (Fig. 3C). CTL blasts efficiently killed also Tag^+^ B6/K-0 targets (Fig. 3), therefore demonstrating that these CTL were able to recognize the endogenously processed and presented SV40 epitope. When cohorts of TRAMP, TRAMPJα18^−/−^ and WT males were immunized after week 10, at which point, tumor-specific CTL tolerance is full-blown [30], a Tag-specific immune response was detected only in splenocytes from vaccinated WT animals (Fig. 3, panels D, E and F, respectively), suggesting that tolerance of Tag-specific CTL proceeded at comparable rates in TRAMP mice bearing or lacking iNKT cells.

**Figure 4:**
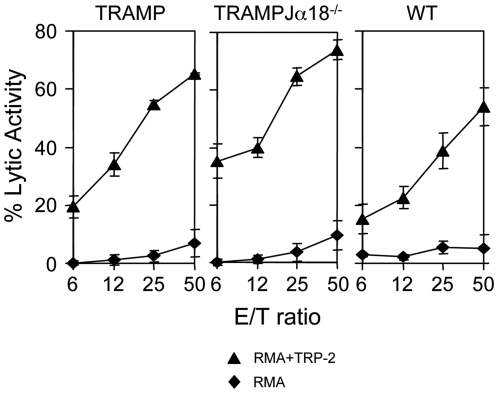
Tolerance in tumor-bearing mice is specific for the PC-related Ag Tag. TRP-2-pulsed DC were injected once i.d. into 16-week old male TRAMP (left panel), TRAMPJα18^−/−^ (middle panel) and WT age- and sex-matched littermates (right panel). After 7 days, animals were killed and their splenocytes were stimulated *in vitro* with the TRP-2 peptide, and tested 5 days later for cytotoxic activity (measured as ^51^Cr release); un-pulsed (black diamonds) or TRP-2-pulsed (black triangles) RMA cells were used as targets. Each panel is representative of at least two independent experiments.

To investigate whether lack of immune response against Tag in aged TRAMP and TRAMPJα18^−/−^ mice reflected a generalized immune suppression or was specific for the TAA, cohorts of TRAMP, TRAMPJα18^−/−^ and WT mice were immunized with DC pulsed with the unrelated and K^b^-restricted melanoma antigen TRP-2. As reported in Fig. 4, all groups of mice generated a comparable TRP-2-specific CTL response, therefore confirming that tolerance in TRAMP and TRAMPJα18^−/−^ mice is restricted to the PC-associated antigen.

**Figure 5:**
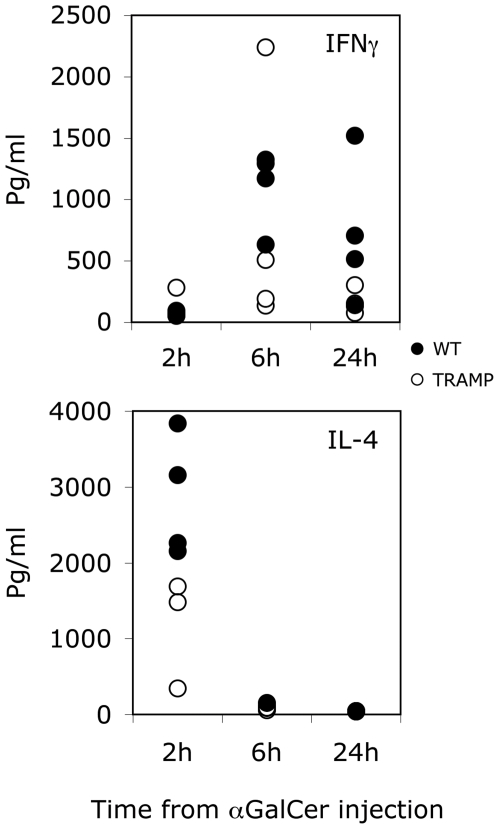
αGal-Cer-mediated release of iNKT-associated cytokines. αGalCer (2 µg/mouse) was administered i.v. to TRAMP (white circles) and WT (black circles) littermates, and blood samples were collected from the tail vein 2, 6 and 24 hours later, and the serum content of IL-4 and IFN-γ was determined by standard ELISA. Values are reported as concentration of the cytokine (pg/ml) in the sera of each animal (3–4/experimental group) analyzed at the indicated time points. Reported data are representative of at least two independent experiments.

Since it has been reported that immunization of mice with the iNKT cell strong agonist antigen αGalCer enhances the T cell response specific for a concomitantly administered antigen, mainly via the NKT cell-mediated licensing of DC function [10], we investigated whether the presence of αGalCer on DC could break Tag-specific CTL tolerance in TRAMP mice. As a preliminary experiment, we compared the functional activity of iNKT cells both in TRAMP and WT C57BL/6 male mice by determining the systemic release of IL-4 and IFNγ after injection of αGalCer i.v. As expected, IL-4 and IFNγ concentration in the serum peaked at 2 and 6 hours after αGalCer injection (Fig. 5), with no statistically significant differences between TRAMP and WT C57BL/6 mice. Furthermore, both frequency and absolute number of splenic and hepatic iNKT cells, determined by αGalCer-mCD1d tetramer staining, were also comparable between the two strain of mice (data not shown). We then immunized fifteen week-old TRAMP mice with DC pulsed either with Tag-IV and αGalCer or with Tag-IV alone. One week later, the presence of Tag-specific CTL in the immunized mice was determined by *in vitro* restimulation of spleen cells with the TAA. A Tag-specific CTL response, however, could not be found in either group of vaccinated mice (data not shown).

Taken together, these data suggest that Tag-specific peripheral CTL tolerance, induced by PC development and progression, is not influenced by iNKT cells, and that iNKT cells exert an as yet undefined role in PC immune surveillance in TRAMP mice.

## Discussion

This study demonstrates that iNKT cells play a relevant role in the immune surveillance of spontaneous prostate adenocarcinoma in TRAMP mice, extending substantially the findings that these cells suppress the onset of spontaneous sarcomas and hematopoietic malignanices [24]. The observation that the lack of iNKT cells correlates with a more aggressive disease in TRAMP mice is consistent with the findings showing that the progression of different types of human cancers, including PC, is accompanied by a significant decrease in iNKT cell number [15], [16], [17], [18]. Thus, iNKT cells appear to play an active role both in mice and in humans in controlling different forms of cancer, ranging from hematological malignancies to sarcoma and carcinoma, arguing for an unconstrained capacity of these cells to survey transformed cells in multiple organs and tissues, through as yet unrecognized mechanisms. Furthermore, our results underscore the relevance of the TRAMP model for deciphering the mechanisms by which the immune system controls solid tumor outgrowth.

iNKT cells display a powerful helper activity for adaptive T as well as B cell responses, exerted mainly via enhancing antigen presenting functions in DC [10], [11], which result in facilitated priming of peptide-specific CD4^+^ and CD8^+^ T cell responses. Furthermore, iNKT cells reduce the immunosuppressive activity of influenza A virus–induced Gr1^+^CD11b^+^/CD15^+^CD11b^+^ myeloid precursors in mice and humans, respectively, by promoting their maturation into functional DC [42]. Given that Gr1^+^CD11b^+^ myeloid precursors share phenotypic and functional characteristics with myeloid-derived suppressor cells (MDSC) [43], iNKT cells might exert their anti-tumor effector functions via modulation of MDSC activity, resulting in a reduced suppression of tumor-specific T cell responses [44]. Such mechanisms could enhance tumor-specific T cell response as well. However, such Gr1^+^CD11b^+^ cells appear defective and may actually suppress iNKT cells in tumor-bearing animals [45]

We did not find evidence that supports a correlation between the presence of iNKT cells in TRAMP mice and either the efficiency of the Tag-specific CTL response or a decreased induction of tolerance toward Tag. Indeed, Tag-specific tolerance appeared at similar rates in TRAMP and TRAMPJα18^−/−^ mice, and could not be broken by immunization with DC pulsed with Tag-IV plus αGalCer. Moreover, we have found that the frequency and function of MDSC as well as CD4^+^CD25^+^Foxp3^+^ regulatory T cells [46] are similar in TRAMP [[29] and Capuano G. et al., manuscript in preparation] and TRAMPJα18^−/−^ mice (data not shown), suggesting that a direct cross-talk between iNKT cells and MDSC or regulatory T cells is not a relevant mechanism in TRAMP mice. Altogether, these data provide indirect evidence that iNKT cells are not involved in controlling the induction of CTL tolerance in TRAMP mice and, consequently, the anti-tumor effector T cell response.

Studies performed *in vitro* have shown that human iNKT can be directly cytotoxic against CD1d^+^ tumor cell targets. The expression of CD1d has been detected on some types of primary human leukemia blasts [47] and on glioma cells [48]. However, the expression of CD1d on mouse tumor cells seems more frequent [49] and our preliminary data suggest that mouse primary PC cells from TRAMP mice express CD1d (data not shown). Nevertheless, it should be taken into consideration that in the absence of strong exogenous ligands such as αGalCer, iNKT exhibit little cytotoxic activity against CD1d^+^ mouse and human tumor cells [47], [50]. Therefore, whether iNKT cells can directly kill tumor cells *in vivo* remains to be established. Interestingly, in this context, human iNKT cells can kill macrophages that are supposed to infiltrate human neuroblastoma in a CD1d-dependent manner [51], providing a mechanistic link between neuroblastoma infiltration by iNKT cells and an improved prognosis [51]. We can therefore speculate that mouse iNKT cells may control PC growth in TRAMP mice by limiting the number or altering the function of tumor associated macrophages (TAM), which play an important role in supporting neoangiogenesis and tumor growth [52] via IFN-γ production.

The definition of the mechanism by which iNKT cells control mouse PC growth warrants future investigation.
